# CD31 ^+^ T‐cells express greater VEGF‐A than CD31 ^−^ counterparts with expression exacerbated with advancing age in male adults

**DOI:** 10.14814/phy2.71057

**Published:** 2026-08-09

**Authors:** Luke Stephen, Graham Wright, David J. Muggeridge, Melanie Leggate, Vignesh Chandrakumar, Mark Ross

**Affiliations:** ^1^ School of Energy, Geoscience, Infrastructure and Society, Institute of Life and Earth Sciences Heriot‐Watt University Edinburgh UK; ^2^ School of Applied Sciences Edinburgh Napier University Edinburgh UK

**Keywords:** age, cardiorespiratory fitness, CD31, T‐cells, VEGF

## Abstract

CD31^+^ T‐cells possess angiogenic properties and have been termed angiogenic T‐cells (T_ANG_) and are altered in number with advancing age. We examined circulating T_ANG_ subsets and VEGF‐A content in young (*n* = 16, 18–30 years) and older (*n* = 16, 50–65 years) males by flow cytometry. Cardiorespiratory fitness ($\dot{V}$ O_2_max) was quantified. Circulating IL‐6 and cytomegalovirus (CMV) serostatus were determined by immunoassays. T_ANG_ contained more VEGF‐A than CD31^−^ T‐cells (CD31^+^: 9374 ± 8587 AU vs. CD31^−^: 8722 ± 8149 AU, *p* = 0.021), also evident in CD4^+^ and CD8^+^ subsets. Older adults possessed fewer CD4^+^ T_ANG_ cells as a proportion of total CD4^+^ T‐cells than younger adults (young: 35% ± 11%; older: 24% ± 9%, *p* = 0.004), and all T_ANG_ subsets from older adults exhibited higher VEGF‐A content than younger adults (CD3^+^: young: 6081 ± 4001 AU; older: 13426 ± 10,945 AU, *p* = 0.019; CD31^+^: young: 6373 ± 3972 AU; older: 15660 ± 12,829 AU, *p* = 0.011; CD8^+^: young: 6335 ± 4029 AU; older: 11216 ± 8056 AU, *p* = 0.043). T_ANG_ cells and VEGF‐A expression were not independently associated with CMV serostatus, IL‐6 or $\dot{V}$ O_2_max. Advancing age is associated with a pathological T_ANG_ phenotype which may contribute to age‐related inflammation.

## 1 INTRODUCTION

T‐cells are a key part of the adaptive immune system, whose primary role is to combat infection (Sun et al., 2023). However, T‐cells also demonstrate various non‐immune functions such as tissue repair and maintenance (Arpaia et al., 2015). These adaptive immune cells are commonly found in secondary lymphoid organs as well as in the circulation. However, T‐cells also reside in, and traffic to, various non‐lymphoid organs such as the lungs, liver (Li et al., 2025), as well as skeletal muscle (Deyhle & Hyldahl, 2018), and therefore likely to contribute to local tissue maintenance.

A specific subset of T‐cells which express the platelet endothelial cell adhesion molecule (PECAM; CD31) have been reported to possess angiogenic properties (Hur et al., 2007; Kushner, Maceneaney, et al., 2010). These angiogenic T‐cells (T_ANG_) have been shown to stimulate proliferation of endothelial progenitor cells (EPC) in vitro as well as promote perfusion recovery after hindlimb ischaemia in a mouse model (Hur et al., 2007) strongly suggesting that they act on endothelial cells to support vascular growth. These cells have strong migratory capacity, to both SDF‐1 (commonly released from injured or ischaemic tissue) and VEGF‐A (Kushner, Maceneaney, et al., 2010). Together these observations suggest that these cells may play an important role in vascular tissue repair.

Aging results in an altered immune system, characterized by inflammation, resulting in increased risk of autoimmune disorders and other chronic illnesses such as coronary artery disease and osteoporosis (Targonski et al., 2007). It is commonly acknowledged that primary indicators of an aging immune system include a marked decline in T‐cell proliferation (Han et al., 2023), and a shift from naïve to differentiated T‐cell phenotype (Bruunsgaard et al., 1999; Fraser & Owen, 2024; Soto‐Heredero et al., 2023). Together, these result in poor vaccine efficacy and adaptive immune response in older adults (Gustafson et al., 2020). There are several reports that T_ANG_ (defined as CD3^+^CD31^+^) cells are lower in older adults compared with younger counterparts (Ross, Ingram, et al., 2018; Ross, Malone, et al., 2018). Moreover, these cells in older adults display a greater senescent profile (Ross, Ingram, et al., 2018), reflective of a higher pro‐inflammatory profile, which is characterized by an increased secretion of inflammatory cytokines IFN‐γ, IL‐6 and TNF‐α (Zhang et al., 2021). In addition, the cell surface expression of a key chemokine receptor involved in cell migration and homing (CXCR4) is also reduced with advancing age (Ross, Malone, et al., 2018). These cells reportedly highly express the potent pro‐angiogenic factor vascular endothelial growth factor‐A (VEGF‐A) (Hur et al., 2007), yet no study has investigated whether VEGF‐A expression in T_ANG_ cells is affected by age, which may partly explain age‐related impairment in angiogenic ability (Rivard et al., 1999).

Interestingly, a high cardiorespiratory fitness (CRF) has been shown to play a role in attenuating the negative effects of aging on T‐cells (Duggal et al., 2018), with individuals displaying higher CRF demonstrating a greater proportion of naïve T‐cells and a decreased proportion of pro‐inflammatory senescent T‐cells (Spielmann et al., 2011). However, a key driver of immunological aging and the associated immune phenotype shift is cytomegalovirus (CMV) serostatus. CMV infection is highly prevalent in older adults and is strongly associated with expansions of highly differentiated, senescent T‐cells (Pawelec et al., 2009), as well as age‐related immune dysfunction (Solana et al., 2012). Despite the established role of CMV in T‐cell immunosenescence, no study to date has investigated whether age, cardiorespiratory fitness, and/or CMV serostatus influences specific T_ANG_ subsets (CD4^+^ vs. CD8^+^), CXCR4^+^ cell numbers, or intracellular VEGF‐A content. Therefore, this study investigated the effect of age, CRF, CMV serostatus and IL‐6 concentrations on total CD3^+^CD31^+^ T‐cells, CD4^+^ and CD8^+^ subsets, CXCR4 expression, and intracellular VEGF‐A expression in humans. As the primary objective of this study was to investigate age‐related differences in angiogenic T‐cell responses, only male participants were included. Aging‐related differences in immune cell populations are influenced by biological sex (Fang et al., 2023; Huang et al., 2021) and as a result, restricting the cohort to a single biological sex minimized additional physiological variability and allowed the effects of age to be examined more directly.

## 2 MATERIALS AND METHODS

### 2.1 Ethical approval

The study was undertaken in accord with the Declaration of Helsinki, and ethical approval was granted by Edinburgh Napier University's Research Ethics and Governance Committee (reference number; 2842425).

### 2.2 Participants and screening

Thirty‐two healthy, non‐obese (BMI < 30 kg·m^2^), non‐smoking male participants aged 18–30 years (*n* = 16) and 50–65 years (*n* = 16) were recruited for this study. All participants gave written informed consent and completed health questionnaires to highlight any general health and cardiovascular concerns before data collection. Participants reported to the Edinburgh Napier University Human Performance Laboratory after an overnight fast, having not exercised for ≥24 h before the visit, having refrained from alcohol consumption the night before, and having not ingested caffeine the morning of the visit. Participants were measured for height and body mass (from this, body mass index (BMI) was calculated). Blood pressure (BP) was measured twice using an automated BP cuff (OMRON HEM‐907, OMRON, Japan) after a 5‐min supine rest. The average of the two blood pressure readings was then recorded.

### 2.3 Assessment of cardiorespiratory fitness

An incremental ramp exercise test to volitional exhaustion was performed to quantify maximum oxygen consumption ($\dot{V}$ O_2_max). The exercise test was performed on a cycle ergometer (Lode Corival, Lode B.V., Netherlands) with simultaneous breath‐ by‐breath gas analysis (Cortex Metalyzer, Germany). Participants performed a warmup for 3‐min, cycling at an initial power output of 100 W for the younger participants and 50 W for the older participants at 60–80 rpm. Following the warm‐up, resistance was increase by 25 W every minute until the participant could no longer maintain 60 rpm or until volitional exhaustion. Heart rate was monitored throughout via heart telemetry (Polar H7 Heart Rate Monitors, Polar, Finland). $\dot{V}$ O_2_max was determined as the average $\dot{V}$ O_2_ of the last 30 s of the exercise test.

Participant characteristics are shown in Table 1.

**TABLE 1:** Participant characteristics (*n* = 32).

	18–30 years (*n* = 16)	50–65 years (*n* = 16)	*p*‐value
Age (years)	**24 ± 2**	**57 ± 4**	**<0.001** *
Height (cm)	**183 ± 7**	**177 ± 7**	**0.010** *
Body Mass (kg)	83 ± 9	84 ± 12	0.668
Body Mass Index (kg·m^2^)	**24 ± 2**	**26 ± 3**	**0.030** *
Systolic Blood Pressure (mmHg)	125 ± 8	131 ± 16	0.249
Diastolic Blood Pressure (mmHg)	81 ± 5	79 ± 8	0.500
$\dot{V}$ O2max (ml·kg·min^−1^)	**39 ± 7**	**29 ± 10**	**0.006** *

*Note*: Values shown are mean ± SD.

* *p* < 0.05 between age groups.

### 2.4 Blood collection

Venepuncture was performed with the participants in a seated, upright position after a 5‐min rest period, prior to the assessment of $\dot{V}$ O_2_max. A 21‐guage needle and collection kit (BD Biosciences, USA) were used for collecting the blood samples. Blood samples were collected into 6 × 10 mL EDTA coated tubes and 1 × 6 mL serum separating tube using the BD Vacutainer Safety‐Lok system (BD Biosciences, USA). Blood samples were used for T‐cell analysis (see Peripheral Blood Mononuclear Cell Separation and Flow Cytometric Quantification of CD31^+^ T‐Cells), serum analysis for cytomegalovirus (CMV) and plasma analysis for IL‐6 concentrations (see Plasma and Serum Analysis for IL‐6 and CMV Serostatus).

### 2.5 Peripheral blood mononuclear cell separation and flow cytometric quantification of CD31 ^+^ T‐cells

Peripheral blood mononuclear cells (PBMC) were isolated with density gradient centrifugation using Lymphoprep™ (Axis‐Shield, USA; cat: AXS‐1114544) as performed previously (Ross, Malone, et al., 2018).

For flow cytometric enumeration of CD31^+^ T‐cells, firstly, 1.0 × 10^6^ mononuclear cells were incubated with monoclonal antibodies targeted to cell surface markers (2 μL of anti‐CD3‐FITC [cat: 981002], anti‐CD4‐BV510 [cat: 317444], anti‐CD8‐PE‐Cy5 [cat: 344770], anti‐CD31‐BV421 [cat: 303124]and anti‐CXCR4‐PE‐Cy7 [cat: 306514]–all Biolegend, USA) for 30 min at 4°C in the dark. The cells were then permeabilised using a permeabilization buffer (BD Cytoperm, BD Biosciences, USA; cat: 554714) and incubated for 20 min at 4°C in the dark. The cells were then washed using a wash buffer (BD Perm/Wash, BD Biosciences, USA; cat: 554714) followed by incubation with 2 μL anti‐VEGF‐A‐PE (Abcam, UK; cat: ab209439) for 30 min at 4°C in the dark. Immediately before flow cytometric enumeration, 500 μL PBS‐BSA was added. T_ANG_ cells (defined as: CD3^+^CD31^+^, CD3^+^CD4^+^CD31^+^, CD3^+^CD8^+^CD31^+^) CXCR4 cell surface expression and intracellular VEGF‐A content were quantified on a 3‐laser flow cytometer (BD FACSCelesta, BD Biosciences). A minimum of 20,000 lymphocyte events were collected per sample. Isotype for CXCR4‐PE‐Cy7 (PE‐Cy77 Mouse IgG2a, Biolegend, USA; cat: 400232), and a negative sample for VEGF‐A‐PE (Abcam, UK) were used as controls to distinguish between positive and negative events, and fully stained non‐permeabilised controls were also used. Compensation was applied to correct for fluorescent spillover between detectors and channels using appropriate compensation beads (BD™ CompBeads, BD Biosciences, USA; cat: 552843) and single stained controls. Although surface marker analyses were completed in all participants, three samples yielded insufficient numbers of analyzable cells following intracellular staining procedures and were therefore excluded from the VEGF‐A analyses.

After data acquisition, data were analyzed using Flow Logic (FlowLogic Version 8.7, Inivai Technologies, Australia). The percentage of lymphocyte subsets of interest was analyzed (CD3^+^CD31^+^ cells, with co‐expression of CXCR4, as well as CD4^+^ and CD8^+^ subsets) and circulating numbers were calculated by multiplying the percentage count as quantified by semi‐automated hematology analyzer (XS‐1000i, Sysmex, Japan). All hematology analyzer data were measured in duplicate and averaged. Expression of VEGF‐A within these subsets was expressed as median fluorescent intensity (MFI).

**FIGURE 1:**
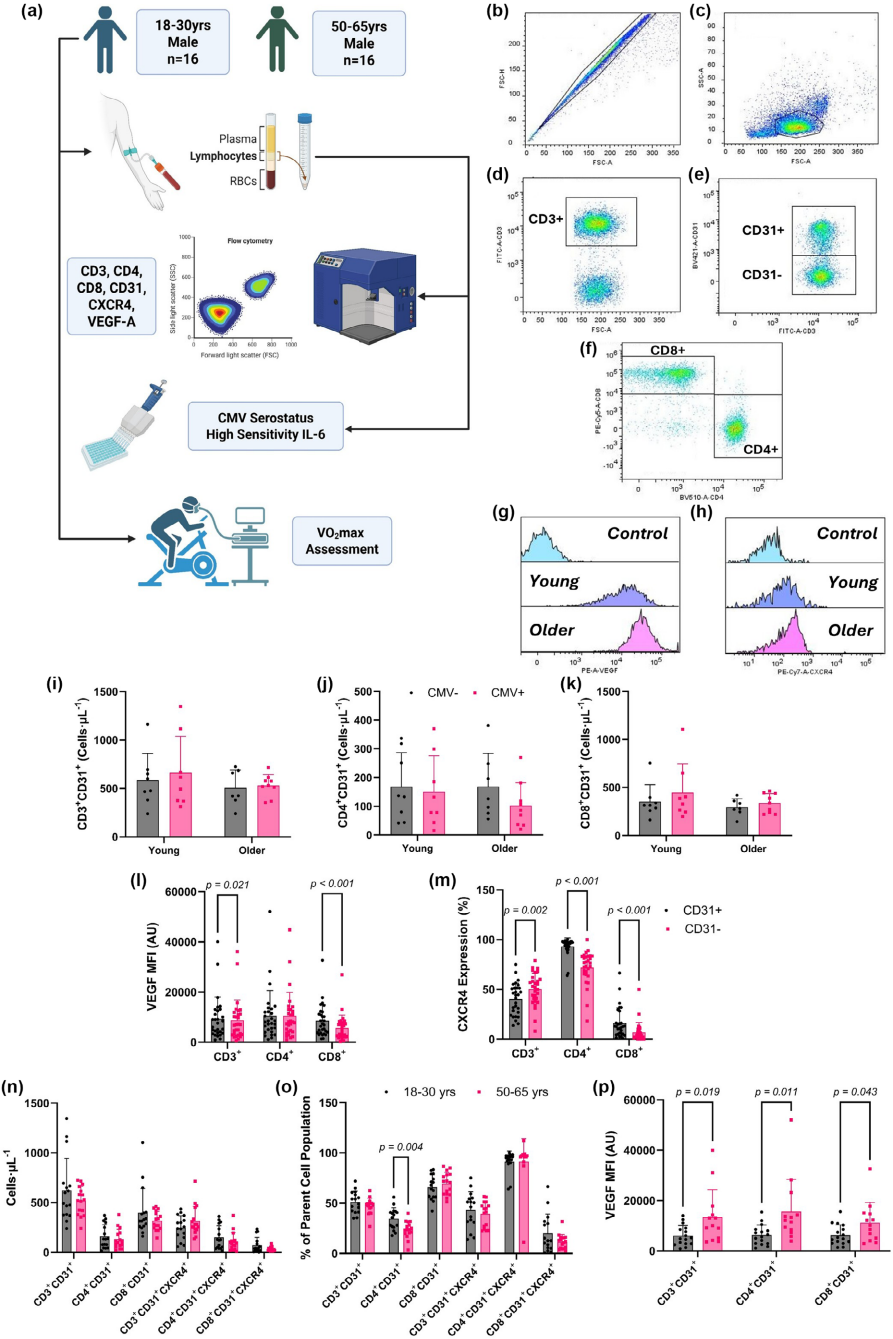
Study design, gating strategies for the flow cytometric quantification of T_ANG_ cells and differences in T_ANG_ cells and VEGF and CXCR4 expression by phenotype and age. (a) Young (18–30 years) and older (50–65 years) adults provided blood samples for quantification of T‐cells, IL‐6 and CMV serostatus, and underwent a graded exercise test to exhaustion for quantifying $\dot{V}$ O_2_max. (b–g) Gating strategy for T_ANG_ cell quantification: First, identification of singlet cells (b), subsequent detection of the lymphocyte population, forward scatter vs. side scatter (FSC vs. SSC); (c), and identification of the CD3^+^ T‐cell population (d) and the CD31^+^ and CD31^−^ subsets (e), and further gating of CD4^+^ population and CD8^+^ populations (f). Finally, the expression of VEGF (g) and CXCR4 (h) were quantified in these cells. (i–k) No effect of CMV serostatus on CD3^+^CD31^+^ (i), CD4^+^CD31^+^ (j) or CD8^+^CD31^+^ (k) T‐cells. (l–n) Analysis for differences in VEGF (l) and CXCR4 (m) between CD31^+^ and CD31^−^ T‐cells. (n–p) Differences in T_ANG_ subsets (N + O) and T_ANG_ VEGF expression (p) between age groups. Values shown are individual data points and mean ± SD.

Flow cytometry gating strategy is shown in Figure 1.

### 2.6 Plasma and serum analysis for IL‐6 and CMV serostatus

After phlebotomy, serum vacutainers were left at room temperature for a minimum of 30 min prior to centrifugation. The vacutainers were subsequently centrifuged at 900 × *g* for 15 min at 4°C, with serum aliquoted and stored at −80°C until analysis. Serum and plasma samples were thawed and used to quantify CMV serostatus (positive, negative) and IL‐6 concentrations (pg·mL^−1^), respectively, using commercially available enzyme‐linked immunosorbent assays (ELISA; Human Anti‐CMV IgG ELISA Kit, Abcam, Cambridge, UK; catalogue number ab108724; Human IL‐6 Quantikine High Sensitivity ELISA Kit, R&D Systems, Minneapolis, MN, USA; catalogue number HS600C). Analysis was performed for both assays using a plate reader (Multiskan FC, Thermofisher, USA), with fluorescence assessed using a 450 nm filter. Samples were analyzed in duplicates, with intra‐assay coefficient of variation of 5.5% and 6.6% for the IL‐6 and CMV assay, respectively. The lower limit of detection for the IL‐6 assay was reported as 0.03 pg·mL^−1^.

### 2.7 Statistical analysis

A Shapiro–Wilk test was used to confirm that the data were normally distributed. Paired *t*‐tests were performed to assess VEGF‐A MFI and CXCR4 expression differences between CD31^+^ and CD31^−^ T‐cells. Subsequently, independent *t*‐tests were performed to assess whether there were any significant differences in circulating T_ANG_ number (CD3^+^CD31^+^, CD4^+^CD31^+^, CD8^+^CD31^+^, and CXCR4^+^ subsets) and T_ANG_ intracellular VEGF‐A expression between young and older adults. Multiple linear regressions were performed to determine the relationship between age and cardiorespiratory fitness and various T_ANG_ subsets, expression of cell surface CXCR4 and intracellular VEGF‐A. Data were analyzed using GraphPad Prism (version 9, Dotmatics, USA) and Jamovi (version 2.7.17; https://www.jamovi.org).

Significance was set at *p* < 0.05. Data are presented as means ± SD unless stated otherwise.

## 3 RESULTS

### 3.1 CD31 ^+^ T‐cells are not influenced by CMV serostatus

Comparisons between CMV seropositive and seronegative individuals demonstrate that there were no statistically significant differences between any CD3^+^CD31^+^ T‐cell subset (proportion or absolute counts), T_ANG_ CXCR4 or VEGF‐A expression (all *p* > 0.05; absolute T_ANG_ data shown in Figure 1).

### 3.2 CD31 ^+^ T‐cells express greater intracellular VEGF content than CD31 ^−^ T‐cells

Our analysis demonstrates that CD31^+^ T‐cells contain greater intracellular VEGF‐A content (defined as VEGF MFI) than CD31^−^ counterparts. This finding persists for total CD31^+^ T‐cells (CD31^+^: 9374 ± 8587 AU vs. CD31^−^: 8722 ± 8149 AU, *p* = 0.021) and CD8^+^CD31^+^ T‐cells (CD31^+^: 8523 ± 6528 AU vs. CD31^−^: 5667 ± 5206 AU, *p* < 0.001). However, no statistically significant difference was found for CD4^+^CD31^+^ T‐cells (*p* = 0.791).

CXCR4 expression was greater in CD31^−^ vs. CD31^+^ T‐cells (CD31^+^: 41% ± 16%; CD31^−^: 49% ± 16%, *p* = 0.011). However, upon further analysis of the CD4^+^ and CD8^+^ subsets, CXCR4 expression was greater in CD4^+^CD31^+^ vs. CD4^+^CD31^−^ cells (93% ± 8%; 72% ± 17%, *p* < 0.001) and similar was observed in the CD8^+^ subset (16% ± 16%; 7% ± 10%, *p* < 0.001).

CXCR4 expression was significantly higher in CD4^+^CD31^+^ T‐cells than CD8^+^CD31^+^ T cells (CD4^+^CD31^+^: 93% ± 8%; CD8^+^CD31^+^: 16% ± 16%, *p* < 0.001). VEGF‐A expression was not different between CD4^+^CD31^+^ and CD8^+^CD31^+^ T‐cells (CD4^+^: 10536 ± 10,054 AU vs. CD8^+^: 8523 ± 6528 AU *p* = 0.125).

Data is shown in Figure 1.

### 3.3 Older adults display reduced proportion of CD4 ^+^ T‐cells expressing CD31 than younger adults

There were no significant differences in CD3^+^CD31^+^ or CD8^+^CD31^+^ T‐cells between young and older adults, either as absolute counts or proportion data (all *p* > 0.05). Older adults did display fewer CD4^+^CD31^+^ cell profiles than younger adults, represented as a proportion of all T‐cells (young: 35% ± 11%; older: 24% ± 9%, *p* = 0.004) but not as absolute counts (young: 159 ± 119 cells·μL^−1^; older: 131 ± 100 cells·μL^−1^, *p* = 0.482).

There were no differences in CXCR4^+^ T_ANG_ counts between the age groups (all *p* < 0.05); however T_ANG_ VEGF‐A MFI was significantly higher in older adults compared to younger adults in all T_ANG_ subsets (CD3^+^CD31^+^: young: 6081 ± 4001 AU; older: 13426 ± 10945 AU, *p* = 0.021; CD4^+^CD31^+^: young: 6373 ± 3972 AU; older: 15660 ± 12829 AU, *p* = 0.009; CD8^+^CD31^+^: young: 6335 ± 4029 AU; older: 11216 ± 8056 AU, *p* = 0.043).

Data for age comparisons is shown in Table 2.

**TABLE 2:** Angiogenic T‐cell number and expression of CXCR4 and VEGF in young (18–30 years) and older (50–65 years) adults (*n* = 32).

	18–30 years (*n* = 16)	50–65 years (*n* = 16)	*p*‐value	*t*‐statistic
CD3^+^CD31^+^				
Cells·μL^−1^	623 ± 322	519 ± 144	0.250	1.174
Proportion (%)	51 ± 11	47 ± 8	0.230	1.225
CXCR4^+^ Cells·μL^−1^	248 ± 119	315 ± 149	0.167	1.425
Proportion (%)	43 ± 18	39 ± 13	0.480	0.715
VEGF MFI	**6081 ± 4001**	**13,426 ± 10,945**	**0.019** *	**2.496**
CD4^+^CD31^+^				
Cells·μL^−1^	159 ± 119	131 ± 100	0.482	0.712
Proportion (%)	**35 ± 11**	**24 ± 9**	**0.004** *	**3.135**
CXCR4^+^ Cells·μL^−1^	149 ± 118	120 ± 97	0.481	0.715
Proportion (%)	91 ± 11	96 ± 3	0.135	1.539
VEGF MFI	**6373 ± 3972**	**15,660 ± 12,829**	**0.011** *	**2.748**
CD8^+^CD31^+^				
Cells·μL^−1^	400 ± 242	318 ± 95	0.215	1.266
Proportion (%)	62 ± 13	68 ± 11	0.386	0.880
CXCR4^+^ Cells·μL^−1^	37 ± 38	28 ± 19	0.061	1.950
Proportion (%)	20 ± 19	11 ± 7	0.109	1.653
VEGF MFI	6660 ± 3946	11,216 ± 8056	0.063	1.942

*Note*: Values shown are mean ± SD.

Abbreviation: MFI, median fluorescence intensity.

* *p* < 0.05 between age groups.

### 3.4 CD8 ^+^ CD31 ^+^ T‐cells influenced by cardiorespiratory fitness, independent of age

The multiple linear regression analyses demonstrated that CD4^+^CD31^+^ T_ANG_ (% of CD4^+^) were inversely associated with age (*r* ^2^ = 0.148, *F* = 5.060, *p* = 0.032), with no other subset demonstrating a statistically significant relationship. After controlling for age, CRF was inversely associated with CD8^+^CD31^+^ T_ANG_ (% of CD8^+^) (*r* ^2^ = 0.201, *F* = 0.750, *p* = 0.011). No other relationships between cardiorespiratory fitness and T_ANG_ subset were observed (all *p* > 0.05).

For other subsets, for both simple, and multiple regression (control for age) revealed no association between circulating T_ANG_ CXCR4 or VEGF‐A expression and cardiorespiratory fitness levels ($\dot{V}$ O_2_max). Summary statistics are displayed in Tables S1 and S2.

### 3.5 Circulating CD31 ^+^ T cells are not independently associated with serum IL‐6 concentrations in young and older adults

Older adults demonstrated a higher circulating level of IL‐6 than younger individuals (young: 0.322 ± 0.142 pg·mL^−1^; older: 0.643 ± 0.421 pg·mL^−1^, *p* = 0.025). Linear regression analysis demonstrated a significant inverse association between IL‐6 concentration and CD4^+^CD31^+^ T‐cells (% of CD4^+^ cells; *r* ^2^ = 0.227, *F* = 6.450, *p* = 0.019). Following adjustment for age, this association was no longer significant, suggesting that age accounted for the relationship between IL‐6 and CD4^+^CD31^+^ T‐cell abundance. No significant unadjusted or adjusted associations were identified for the remaining T‐cell subsets, including CXCR4‐ and VEGF‐A‐expressing cells (all *p* > 0.05). Summary statistics are displayed in Tables S3 and S4.

## 4 DISCUSSION

The study's main findings are that CD31^+^ T‐cells express greater VEGF‐A, a key angiogenic growth factor, than CD31^−^ T‐cells. Paradoxically, whilst CD31^+^ T‐cell numbers did not differ between age groups, VEGF‐A expression was elevated in CD31^+^ T‐cells from older adults, suggesting a higher basal inflammatory profile. In addition, we demonstrated that these angiogenic T‐cells also have greater CXCR4 expression than CD31^−^ T‐cells, suggestive of a greater migratory function to SDF‐1, which is elevated in tissue injury (Ratajczak et al., 2004).

VEGF‐A is a highly pro‐angiogenic growth factor (Wagner, 2011), linked to both hypoxia‐ and exercise‐induced angiogenesis (Ross et al., 2023; Thom et al., 2014; Wagner, 2011). Various cell types produce and secrete VEGF (Stepanova et al., 2007) including neutrophils (Gong & Koh, 2010), monocytes/macrophages (Stepanova et al., 2007), endothelial cells (Guangqi et al., 2012), and skeletal muscle cells (Delavar et al., 2014; Ryan et al., 2006). In the 1980s, several studies demonstrated that T‐cells produce VEGF, known then as vascular permeability factor (Heslan et al., 1986; Tomizawa et al., 1985). In 2007, a study by Hur et al. (2007) demonstrated that a specific subset of T‐cells that express CD31 were required for optimal in vitro growth of endothelial progenitor cells, with subsequent studies demonstrating a pro‐angiogenic phenotype of these CD31^+^ T‐cells, with work by Kushner, Maceneaney, et al. (2010) observing greater migration to VEGF and SDF‐1 by CD31^+^ vs. CD31^−^ T‐cells. Our study further emphasizes that these CD31^+^ T‐cells likely possess greater angiogenic function than CD31^−^ T‐cells through enhanced VEGF‐A production and greater CXCR4 expression albeit in CD4^+^ and CD8^+^ subsets only. Interestingly, we observed lower CXCR4 expression in CD3^+^CD31^+^ T‐cells as the parent population, likely driven by non‐CD4^+^ or CD8^+^ cells such as γδ T‐cells (Wang et al., 2025), however, these were not quantified in this study, and future work should include analysis of CD31^+^ expression and pro‐angiogenic activity of such cells, as these cells are good candidates for cellular therapies, due to the lower risk of graft versus host disease (Revesz et al., 2024).

Older adults display a lower number of circulating CD31^+^ T‐cells compared to younger adults (Hur et al., 2007; Kushner, Weil, et al., 2010; Ross, Ingram, et al., 2018; Ross, Malone, et al., 2018), with these cells also demonstrating impaired migration, increased apoptosis susceptibility, reduced telomerase activity (Kushner, Weil, et al., 2010), and greater senescence (Ross, Ingram, et al., 2018) compared to younger adults. Together, these findings suggest impaired angiogenic activity of these putative angiogenic T‐cells (T_ANG_), however, our data in this study show that T_ANG_ cells from older adults possess greater VEGF‐A content than younger adults. Rather than this indicating a greater angiogenic capability in these T‐cells, we suggest this excess unstimulated VEGF‐A production by these cells in older adults is indicative of a chronic inflammatory state. Indeed, it is known that excessive VEGF‐A signaling is associated with several inflammatory states, including diabetic retinopathy (Ahmad & Nawaz, 2022; Gu et al., 2020), cancer (Ahmad & Nawaz, 2022; Freeman et al., 1995) and rheumatoid arthritis (Foster et al., 2009). There is evidence of excess VEGF‐A production by T‐cells (CD4^+^) in type 1 diabetics (Marek et al., 2010), as well as in patients with chronic obstructive pulmonary disease (Mikko et al., 2009), which may be a key contributor to tissue inflammation in these states. The data in our study suggest that whilst CD31^+^ T‐cells contain more VEGF‐A than CD31^−^ T‐cells, which likely contributes to the vascular phenotype of these T‐cells, may also, with advancing age, contribute to T‐cell mediated inflammation in age‐related disease (Bektas et al., 2017; Macaulay et al., 2013; Yu et al., 2016). We did not co‐stain for CD28 to perform sub‐analysis on VEGF‐A production in CD28^null^, senescent‐associated, T_ANG_, with these being elevated in older adults (Ross, Ingram, et al., 2018) and themselves containing more intracellular pro‐inflammatory cytokines (IL‐6, TNF‐α and IFN‐γ) compared to CD28^+^ T_ANG_ cells (Zhang et al., 2021), may be the source of the excess VEGF‐A, rather than all T_ANG_ cells per se. Therefore, as a result of our data and those presented in this discussion, the higher VEGF‐A content of T_ANG_ cells from older adults presents a propensity for pathological angiogenesis and chronic inflammation, and future studies are needed to identify source of the elevated VEGF‐A, and whether this is associated or contributes to inflammatory conditions.

Our subsequent analyses investigated whether cardiorespiratory fitness was associated with our T_ANG_ measures. Cardiorespiratory fitness, which is associated with altered T‐cell phenotypes in several reports (Spielmann et al., 2011) had limited influence on these T_ANG_ cells, with only CD8^+^ T_ANG_ cells demonstrating a significant relationship with cardiorespiratory fitness. Initially there was a negative relationship between CD8^+^ T_ANG_ cells and $\dot{V}$ O_2_max, which is surprising, given CD31 is a marker of recent thymic emigrants, and previous work has demonstrated that $\dot{V}$ O_2_max is positively associated with naïve CD8^+^ T‐cells (Spielmann et al., 2011), which are expected to be more recent emigrants from thymic development than differentiated, effector T‐cells. However, after correcting for age, as our older adults possessed significantly lower levels than their younger counterparts, and previous evidence that aging is associated with a decline in $\dot{V}$ O_2_max (Rogers et al., 1990), there was no significant relationship, indicating that fitness per se, did not influence this subset. Interestingly, cardiorespiratory fitness was positively associated with CD8^+^ T_ANG_ CXCR4 expression which remained significant after controlling for age, suggesting that regular exercise, or physical activity, may influence T_ANG_ migratory potential. We did not control for CMV serostatus, as our analysis demonstrated that cell number and various measures of T_ANG_ subsets were not influenced by CMV serostatus. Our data also shows that older adults displayed a higher BMI than younger adults, and so may be a confounding variable in our analysis. However, after controlling for age, BMI was not associated with any T_ANG_ subset or measure. Further studies are required to elucidate the direct effects of exercise and physical activity interventions on these cells, using in vitro and in vivo studies to determine the potential of these cells to migrate to tissue injury.

Interestingly, our T_ANG_ cellular data was not independently associated with plasma levels of the pro‐inflammatory cytokine IL‐6, which is in contrast to previous published works (Zhang et al., 2021). The primary reason for this discrepancy between these studies is the lack of CD28 phenotyping of our T_ANG_ cells, as Zhang et al. (2021) observed a positive relationship between circulating IL‐6 and CD28^null^CD4^+^ T_ANG_ cells, but did not present data on the parent population (e.g. CD4^+^ T_ANG_). The senescent subset of T_ANG_ cells produce more proinflammatory cytokines (IL‐6, TNF‐α, IFN‐γ) (Zhang et al., 2021) which may contribute to elevated circulating inflammatory milieu with advancing age, however, we cannot determine this from our data, which is a limitation of our study. Also, IL‐6 was the only pro‐inflammatory cytokine measured in this study, and so other age‐related inflammatory factors, such as C‐reactive protein (CRP) and TNF‐α, may, in future, provide more clarity on relationship between immune cell subsets, age and inflammation. It is clear that a more in‐depth phenotyping strategy is required to fully elucidate the impact of inflammation, age, exercise, cardiorespiratory fitness on these T‐cells.

### 4.1 Limitations

As noted above, this study is not without its limitations. Firstly, the flow cytometric phenotypic strategy for this study omitted CD28, thus preventing our ability to determine VEGF‐A content between senescent and non‐senescent T_ANG_ cells to better understand the relationship between these cells and advancing age. Secondly, while CMV serostatus was included as a potential confounding factor, the study was not specifically powered to investigate CMV‐related differences in T_ANG_ cell populations. The relatively small sample size within the age‐stratified CMV subgroups may have limited our ability to detect effects of CMV serostatus on these cell populations. Thirdly, the participant pool was wholly male, and therefore the findings cannot be generalizable to female populations. Future studies are planned to examine how aging and menopause influence T_ANG_ populations, which may yield deeper mechanistic insights into the relationship between menopausal transition and vascular inflammation.

### 4.2 Conclusion

This study is the first to demonstrate that CD31^+^ putative angiogenic T‐cells (T_ANG_) possess greater VEGF‐A content and CXCR4 expression than CD31^−^ T‐cells, suggestive of a greater angiogenic capacity. However, older adults display an excessive T_ANG_ VEGF‐A profile, which is likely to reflect a shift from physiological to pathological angiogenesis and inflammation with advancing age. Cardiorespiratory fitness was not associated with T_ANG_ VEGF‐A content but was positively associated with CD8^+^ T_ANG_ CXCR4 expression, indicating that T_ANG_ migratory capacity (a key function of such cells in angiogenesis) is higher in those with a higher $\dot{V}$ O_2_max. Overall, these findings show that aging drives a distinct shift toward a more inflammatory and dysregulated T_ANG_ phenotype, marking these cells as sensitive indicators of immune–vascular aging.

## AUTHOR CONTRIBUTIONS

**Luke Stephen:** Conceptualization; data curation; formal analysis; investigation; methodology. **Graham Wright:** Conceptualization; funding acquisition; methodology; supervision. **Melanie Leggate:** Methodology; supervision. **David J. Muggeridge:** Methodology; supervision. **Vignesh Chandrakumar:** Investigation. **Mark Ross:** Conceptualization; data curation; formal analysis; funding acquisition; investigation; methodology; project administration; supervision; visualization.

## FUNDING INFORMATION

This work was supported by funds from the Research Excellence Grant by Edinburgh Napier University.

## CONFLICT OF INTEREST STATEMENT

The authors declare no conflicts of interest.

## Supporting information


**Table S1:** Linear regression analysis investigating the influence of age and cardiorespiratory fitness on CD31^+^ T‐cell number (*n* = 32).
**Table S2:** Linear regression analysis investigating the influence of age and cardiorespiratory fitness on CD31^+^ T‐cell CXCR4 and VEGF‐A expression (*n* = 32).
**Table S3:** Linear regression analysis investigating the influence of IL‐6 on CD31^+^ T‐cell number (*n* = 32).
**Table S4:** Linear regression between CD31^+^ T‐cell CXCR4, VEGF‐A expression and IL‐6 (*n* = 32).

## Data Availability

Data will be made available upon reasonable request.
