# Supplementary material for: CD31 + T‐cells express greater VEGF‐A than CD31 − counterparts with expression exacerbated with advancing age in male adults
Source: Physiol Rep. 2026 Aug 9;14(15):e71057. doi: 10.14814/phy2.71057 (PMC13454554; doi:10.14814/phy2.71057)
Supplement: Supplementary file 1 [file PHY2-14-e71057-s001.docx]

**Supplementary Table 1.** Linear Regression Analysis Investigating the Influence of Age and Cardiorespiratory Fitness on CD31⁺ T-Cell Number (n=32).

| **T_ANG_ Subset** | **Model** | **R^2^** | ***F* - Value** | ***p* - Value** | **R^2^ Change** | ***F*-Change** | **Change *p* - Value** |
| --- | --- | --- | --- | --- | --- | --- | --- |
| CD3^+^CD31^+^ | 1 | 0.030 | 0.881 | 0.356 | **-** | - | - |
|  | 2 | 0.075 | 1.132 | 0.337 | 0.045 | 1.370 | 0.251 |
| CD3^+^CD31^+^ (% of CD3^+^) | 1 | 0.022 | 0.664 | 0.664 | - | - | - |
|  | 2 | 0.120 | 1.905 | 0.168 | 0.097 | 3.100 | 0.089 |
| CD4^+^CD31^+^ | 1 | 0.001 | 0.020 | 0.888 | **-** | - | - |
|  | 2 | 0.004 | 0.052 | 0.949 | 0.003 | 0.085 | 0.773 |
| CD4^+^CD31^+^ (% of CD4^+^) | **1** | **0.148** | **5.060** | **0.032*** | - | - | - |
|  | 2 | 0.153 | 2.53 | 0.097 | 0.005 | 0.158 | 0.694 |
| CD8^+^CD31^+^ | 1 | 0.055 | 1.690 | 0.204 | - | - | - |
|  | 2 | 0.107 | 1.670 | 0.207 | 0.051 | 1.610 | 0.215 |
| CD8^+^CD31^+^ (% of CD8^+^) | 1 | 0.047 | 1.440 | 0.239 | - | - | - |
|  | **2** | **0.249** | **4.630** | **0.018*** | **0.201** | **7.500** | **0.011*** |

*Model 1- Age*

*Model 2- Age and* $\dot{V}$*O_2_max*

**p < 0.05.*

**Supplementary Table 2.** Linear Regression Analysis Investigating the Influence of Age and Cardiorespiratory Fitness on CD31⁺ T-Cell CXCR4 and VEGF-A Expression (n=32).

| **T_ANG_ Subset** | **Model** | **R^2^** | ***F* - Value** | ***p* - Value** | **R^2^ Change** | ***F - Change*** | **Change *p - Value*** |
| --- | --- | --- | --- | --- | --- | --- | --- |
| CD3^+^CD31^+^CXCR4^+^ (%) | 1 | 0.005 | 0.133 | 0.718 | - | - | - |
|  | **2** | 0.136 | 2.209 | 0.129 | **0.132** | **4.270** | **0.048*** |
| CD3^+^CD31^+^ VEGF MFI | **1** | **0.173** | **5.640** | **0.025*** | - | - | - |
|  | 2 | 0.186 | 2.960 | 0.069 | 0.013 | 0.411 | 0.527 |
| CD4^+^CD31^+^CXCR4^+^ (%) | 1 | 0.110 | 3.320 | 0.080 | **-** | - | - |
|  | 2 | 0.156 | 2.410 | 0.110 | 0.047 | 1.45 | 0.240 |
| CD4^+^CD31^+^ VEGF MFI | **1** | **0.204** | **6.900** | **0.014*** | **-** | **-** | **-** |
|  | **2** | **0.212** | **3.500** | **0.045*** | 0.008 | 0.273 | 0.606 |
| CD8^+^CD31^+^CXCR4^+^ (%) | 1 | 0.128 | 3.950 | 0.057 | **-** | - | - |
|  | 2 | 0.145 | 2.200 | 0.131 | 0.017 | 0.523 | 0.476 |
| CD8^+^CD31^+^ VEGF MFI | 1 | 0.159 | 3.590 | 0.073 | **-** | **-** | **-** |
|  | 2 | 0.168 | 1.810 | 0.192 | 0.009 | 0.184 | 0.673 |

*Model 1- Age*

*Model 2- Age and* $\dot{V}$*O_2_max*

**p < 0.05. T_ANG_ CXCR4 data presented as proportion data only. MFI- Median Fluorescence Intensity.*

**Supplementary Table 3.** Linear Regression Analysis Investigating the Influence of IL-6 on CD31⁺ T-Cell Number (n=32).

| **T_ANG_ Subset** | **Model** | **R^2^** | ***F* - Value** | ***p* - Value** |
| --- | --- | --- | --- | --- |
| CD3^+^CD31^+^ | 1 | 0.000 | 0.011 | 0.918 |
|  | 2 | 0.012 | 0.251 | 0.622 |
| CD3^+^CD31^+^ (% of CD3^+^) | 1 | 0.010 | 0.225 | 0.640 |
|  | 2 | 0.010 | 0.208 | 0.653 |
| CD4^+^CD31^+^ | 1 | 0.040 | 0.924 | 0.347 |
|  | 2 | 0.054 | 1.209 | 0.284 |
| CD4^+^CD31^+^ (% of CD4^+^) | **1** | **0.227** | **6.450** | **0.019*** |
|  | 2 | 0.136 | 3.702 | 0.068 |
| CD8^+^CD31^+^ | 1 | 0.001 | 0.020 | 0.889 |
|  | 2 | 0.040 | 0.951 | 0.341 |
| CD8^+^CD31^+^ (% of CD8^+^) | 1 | 0.105 | 2.580 | 0.123 |
|  | 2 | 0.018 | 0.461 | 0.505 |

*Model 1- unadjusted*

*Model 2- adjusted for age*

**p < 0.05.*

**Supplementary Table 4.** Linear Regression between CD31^+^ T-Cell CXCR4, VEGF-A Expression and IL-6 (n=32).

| **T_ANG_ Subset** | **Model** | **R^2^** | ***F* - Value** | ***p* - Value** |
| --- | --- | --- | --- | --- |
| CD3^+^CD31^+^CXCR4^+^ (%) | 1 | 0.007 | 0.144 | 0.708 |
|  | 2 | 0.000 | 0.002 | 0.968 |
| CD3^+^CD31^+^ VEGF MFI | 1 | 0.002 | 0.031 | 0.863 |
|  | 2 | 0.067 | 1.630 | 0.218 |
| CD4^+^CD31^+^CXCR4^+^ (%) | 1 | 0.036 | 0.702 | 0.412 |
|  | 2 | 0.003 | 0.060 | 0.809 |
| CD4^+^CD31^+^ VEGF MFI | 1 | 0.001 | 0.001 | 0.971 |
|  | 2 | 0.057 | 1.410 | 0.251 |
| CD8^+^CD31^+^CXCR4^+^ (%) | 1 | 0.004 | 0.092 | 0.764 |
|  | 2 | 0.048 | 1.340 | 0.261 |
| CD8^+^CD31^+^ VEGF MFI | 1 | 0.000 | 0.009 | 0.927 |
|  | 2 | 0.041 | 0.889 | 0.358 |

*Model 1- unadjusted*

*Model 2- adjusted for age,*

*T_ANG_ CXCR4 data presented as proportion data only. MFI- Median Fluorescence Intensity.*
